# Chromatin and epigenetic features of long-range gene regulation

**DOI:** 10.1093/nar/gkt499

**Published:** 2013-06-13

**Authors:** Nathan Harmston, Boris Lenhard

**Affiliations:** ^1^MRC Clinical Sciences Centre, Faculty of Medicine, Imperial College, London W12 0NN, UK, ^2^Institute of Clinical Sciences, Faculty of Medicine, Imperial College, London W12 0NN, UK and ^3^Department of Informatics, University of Bergen, Thromøhlensgate 55, N-5008 Bergen, Norway

## Abstract

The precise regulation of gene transcription during metazoan development is controlled by a complex system of interactions between transcription factors, histone modifications and modifying enzymes and chromatin conformation. Developments in chromosome conformation capture technologies have revealed that interactions between regions of chromatin are pervasive and highly cell-type specific. The movement of enhancers and promoters in and out of higher-order chromatin structures within the nucleus are associated with changes in expression and histone modifications. However, the factors responsible for mediating these changes and determining enhancer:promoter specificity are still not completely known. In this review, we summarize what is known about the patterns of epigenetic and chromatin features characteristic of elements involved in long-range interactions. In addition, we review the insights into both local and global patterns of chromatin interactions that have been revealed by the latest experimental and computational methods.

## INTRODUCTION

Gene regulation during differentiation and development is responsible for the precise coordination of processes, which determine cell fate and how the anatomical plan develops (1). Research in the past couple of decades has shed light on the regulation of some of the genes with the most complex expression patterns (2). The availability of the full sequences from multiple metazoan genomes, as well as an increasing number of high-throughput methods for detecting regulatory elements and monitoring their activity, has revolutionized our understanding of gene regulation. Much of the data produced by large-scale efforts to identify functional elements in the genome, such as ENCODE (3) and modENCODE (4), provide information on the epigenetic state and accessibility of chromatin along the entire genome in various, selected, biological contexts. These data sets contain information about the state of individual regulatory elements (5,6), providing the possibility to add a dynamic, functional layer to studies of regulation at the genomic level.

The various types of regulatory elements in the genome integrate and interpret information from a multitude of regulatory signals to tightly control the spatiotemporal expression of important developmental factors (7). Some genes are regulated by dozens, possibly hundreds, of distal regulatory elements found in large, megabase-sized regions surrounding them (8,9). The extreme distances between regulatory elements and their target genes open a series of questions about an additional aspect of long-range regulation: what is the mechanism by which these elements specifically select and communicate with their target genes, while preventing unwanted promiscuous communication with other genes in the region?

In this review, we give an overview of the current knowledge regarding regulatory elements involved in regulating gene expression, and how these elements communicate with each other during metazoan development. We discuss how the system of interactions between transcription factors (TFs), histone modifications and the 3D organization of chromatin result in the ability to generate complex spatiotemporal expression patterns.

## ELEMENTS OF TRANSCRIPTIONAL CONTROL IN VERTEBRATES

### Promoters and proximal elements

A promoter is the genomic region overlapping the transcription start sites (TSSs) of a gene. Because of the precision with which TSSs can be identified, especially using CAGE technology (10), this class of regulatory elements has been well studied in comparison with other classes. A promoter is typically described as comprising of two elements: a core promoter and a proximal (regulatory) region. The core promoter is the region near the TSS (including the TSS itself), required for the initiation of transcription and the recruitment of RNA polymerase (Pol) II at the TSS. The proximal promoter is defined as the region immediately upstream of the core promoter, and it typically contains several TF-binding sites (TFBSs), which serve as context-specific regulatory inputs to the core promoter (11).

The architecture of a gene’s promoter relates to both the function of the gene and how it is regulated. It has been proposed that there are at least three main functional classes of PolII promoters in metazoa (12). Type I promoters correspond to genes that display tissue-specific expression patterns, feature a sharp TSS with disordered nucleosomes and are not located close to CpG islands. Ubiquitously expressed genes typically have a Type II promoter. These promoters are located near CpG islands and have a broad TSS distribution with ordered nucleosomes. The promoters of developmentally regulated genes, Type III, typically feature a sharper TSS distribution than Type II promoters, are associated with repression by Polycomb proteins and typically have long and/or multiple CpG islands that extend into the body of the gene. We will discuss these classes further in the context of the differences in their epigenomic and histone properties and their propensity towards forming long-distance interactions. In addition to Type III promoters, developmentally regulated genes, such as developmental TFs, cell adhesion proteins and axon guidance mediators, have loci-specific features that set them aside from other genes (13).

### The action of distal *cis*-acting elements

The regulatory content and architecture of the promoters is insufficient for explaining the diversity observed in gene expression patterns, especially the complex patterns of genes whose products are master regulators of development and differentiation. Most of the regulatory content of a metazoan genome lies outside of proximal promoters (14–16); enhancers are the most common and best understood subset of these elements (17). These sequences are up to several hundred base pairs in length and consist of multiple binding sites for many different TFs and chromatin regulators. The combinations of TFs bound at these elements permit the transcriptional control of their target gene in a dosage, spatial and temporal-specific manner. Traditionally, they are thought to act independently of both the distance and orientation relative to the promoter (18), although some have challenged this assumption (19).

Based on the existence of cooperativity between bound TFs and the spacing and order of TFBS within an enhancer (often referred to as an enhancer grammar), studies of *cis*-regulatory elements have found that the arrangement of TFBSs can be classified into one of three distinct architectures (20). The enhanceosome (21) architecture requires that the TFBSs follow a strict order and spacing to allow cooperative interactions between both neighbouring proteins and the bound chromatin. In the billboard model of enhancer function (22), TFs are recruited independently to the enhancer; therefore, the spacing and orientation of TFBSs within the enhancer are not important. An enhancer architecture that does not require a well-defined grammar but allows for cooperativity between TFs, called the TF collective model, has been proposed during studies of mesodermal specification (23). This model allows for turnover of the TFBSs within an enhancer while not affecting its activity.

Studies of the sparkling (*spa*) enhancer of *Pax2* in *Drosophila* (24) have revealed that this enhancer seems to have features indicative of both the billboard and enhanceosome model. Rearrangements of its constitutive TFBSs result in the cell-type specificity of this enhancer being altered, suggesting that the structure of this enhancer is constrained by a number of short-range interactions between bound TFs, while still remaining evolutionarily labile (25). This study also identified a specific sequence at the 5′-end of the enhancer, which was necessary to enhance reporter gene transcription from a distance.

There does not seem to be a limitation to the location of enhancers relative to their target genes (26): in addition to intergenic regions (downstream as well as upstream), they can be located within the introns of the gene they regulate (27), within the introns of neighbouring genes (28), or in intergenic regions beyond neighbouring genes (29). Enhancers can communicate with their target promoter over large distances and over intermediate bystander genes: an enhancer of *Shh*, which is important for limb development, is located within an intron of the *LMBR1* gene situated 1 Mb away (30), with the gene *Rnf32* positioned between. Recently, it has been reported that sequences of coding exons can also act as enhancers for their own (31) or neighbouring genes (32,33). Even though the majority of enhancers are in *cis* to their target genes, and stay in *cis* through large-scale genomic events, such as whole-genome duplication (9,34), there is a handful of documented, but poorly understood, cases of *trans*-enhancers that activate the transcription of genes located on different chromosomes (35).

Recently, it has been suggested that genes important for cell identity are regulated by clusters of adjacent enhancers (36,37). These super-enhancers are bound by mediator, are much larger than normal enhancers, have a high density of TFBSs and binding sites for important TFs involved in regulating cell identity. These elements seem to be extremely sensitive to perturbations, i.e. inhibition of *BRD4* in cancer cells was found to completely ablate its binding to a super-enhancer of *MYC*, leading to a significant decrease in the amount of *MYC* expression (37). Fragments of these elements were found to drive high levels of reporter gene expression. These findings suggest that the TFs bound to these elements are highly co-operative. However, it remains to be shown whether these domains function as a discrete unit or merely reflect enhancers that are clustered together for other reasons, e.g. chromatin accessibility or redundancy.

### Promoter: enhancer interaction

There are several models of how distal elements communicate with their target promoters. In the tracking model, the transcription-initiating complex bound to an enhancer moves along the DNA until it reaches a promoter and initiates transcription (38); this model is highly unlikely to account for interactions between promoters and enhancers at megabase distances. In the facilitated tracking model, the tracking movement causes the chromatin to form a loop between the enhancer and promoter. The linking model proposes that enhancers and promoters communicate via intervening linking proteins, whose binding is mediated by enhancer activity (39). However, the simplest model with the most supporting evidence is that there is a direct physical interaction between enhancers and promoters brought about by chromatin looping (40). The mechanisms responsible for mediating this looping are not completely understood at this time, although cohesin and the CCCTC-binding factor (CTCF) are thought to play a prominent role (41).

## EVOLUTIONARY FEATURES OF *CIS*-ACTING ELEMENTS OF DEVELOPMENTAL REGULATORY GENES

A subset of non-coding elements is highly conserved across vertebrates, in some cases being more conserved than the exons of genes that encode perfectly conserved polypeptides. The level of conservation of these sequences (42), their location within vertebrate genomes (43) and their distribution throughout the vertebrate lineage (44) suggested that these were candidates for regulatory elements important in the early stages of vertebrate development. A subset (>50%) of these conserved non-coding elements (CNEs) has been found to function as enhancers *in vivo* (44,45). The probability that a conserved sequence has enhancer activity is related to its level of conservation and the density of other conserved sequences in the surrounding locus (46,47). However, even developmental enhancers cannot always be identified using DNA sequence conservation methods (48), and their function can be conserved even when their sequence is not (49–51).

Even though many CNEs can function as enhancers, it is puzzling that at least in some cases, deletion of large clusters of CNEs yields viable mice (52) with no obvious deleterious phenotypic changes. The suggested interpretations for this are that some of these elements may be redundant (53), are essential only under specific conditions not found in a laboratory or only have phenotypes that are detectable over many generations.

Since their original discovery in vertebrate genomes, CNEs were found around orthologous genes in other metazoa (54) and recently in plants (55,56). With extremely rare exceptions (26), none of these CNEs in *Drosophila* or *Caenorhabditis elegans* are similar at sequence level to any of the vertebrate CNEs, suggesting the existence of equivalent elements in the common ancestor followed by parallel, slow turnover in independent lineages.

**Figure 1.**
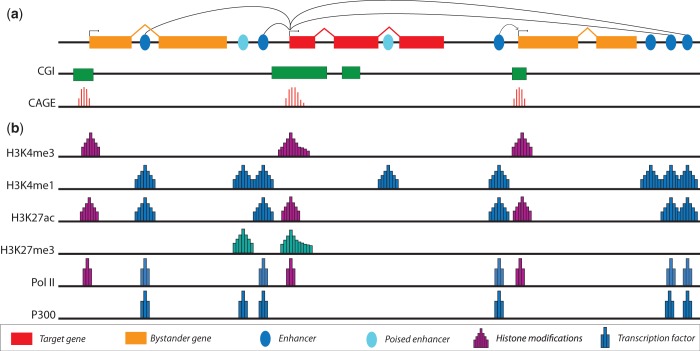
(**a**) The genomic regulatory block model of transcriptional regulation. Genes involved in the regulation of developmental processes are themselves regulated by enhancers located in a variety of locations, both upstream and downstream. Developmentally regulated genes tend to have CpG islands (CGIs) that overlap with its promoter and extend into the body of the gene. Genes with Type II and III promoters typically feature a broad TSS distribution, as detected by CAGE. (**b**) Regulatory elements within the genome can be identified by distinct patterns of histone modifications and TF binding. Promoters are enriched for H3K4me3, with active promoters showing evidence of PolII binding. The presence of the repressive mark at promoters and depletion of H3K4me3 is associated with inactive repressed promoters. Promoters having both H3K4me3 and H3K27me3 are termed bivalent promoters; they are repressed but poised for activation. Both poised and active enhancers are marked by the histone modification H3K4me1 and show depletion of H3K4me3. In addition, active enhancers are marked by H3K27ac and the binding of P300, whereas poised enhancers lack H3K27ac and may be marked by H3K27me3.

Long-range regulation imposes constraints on the organization of the genome (57) and its evolution (58). In *Drosophila* and vertebrates, developmental genes are associated with arrays of enhancers and CNEs (9,54). Maintaining the correct patterns of gene expression requires that regulatory elements are kept in *cis* with their target genes. This has led to the maintenance of large regions of conserved synteny over large evolutionary distances (9,54,59), referred to as genomic regulatory blocks (GRBs). These GRBs can extend over large distances and often span large-genomic regions of low-gene density, called gene deserts, or encompass one or more bystander genes in addition to the target gene (Figure 1). Misregulation of GRB target genes has been implicated in developmental disorders and abnormal phenotypes. Mutations in the gene deserts flanking GRB target genes [e.g. SOX9 (60) and PAX6 (49)] are involved in developmental disorders (61). More generally, mutations in distal regulatory elements have been implicated in a variety of diseases, including cancer (62), type II diabetes (63) and dyslexia (64). It is thought that variation within CNEs may have a role in a number of common human disorders (65), and it may help to explain why a large proportion of disease associated single-nucleotide polymorphisms identified using genome-wide association studies (GWAS) are located within non-coding regions (66,67).

## FUNCTIONAL CHARACTERIZATION OF LONG-RANGE REGULATORY ELEMENTS

The enhancer potential of a putative regulatory sequence is determined by its ability to induce expression in reporter gene assays (68), where enhancer activity is visualized or quantitated based on the activity of a reporter gene. However, induction of reporter gene expression is often an inadequate proxy for the activities of regulatory elements *in vivo*: these elements can be active during narrow windows of development, the host cell could lack the cofactors necessary for enhancer activation, or the sequence context of the reporter gene can be too different from its native location. Historically identifying and validating enhancers has been a laborious process, although new high-throughput methods for interpreting enhancer function are now being developed (69–71).

Enhancer trap systems can be used to identify the genes and loci responsive to native enhancers in the genome (72). In *Drosophila*, enhancer traps have found that the gene closest to the insertion site is the gene whose expression pattern tends to be followed in a majority of cases (73). However, in vertebrates, this is often not the case (28), suggesting that the mechanisms of long-range enhancer:promoter communication get more complicated as distances between promoters and enhancers increase.

## EPIGENETIC FEATURES OF REGULATORY ELEMENTS

The histone code hypothesis postulates that distinct combinatorial patterns of histone modifications are responsible for specifying function (74); reviewed in (75). Different classes of elements are marked by distinct patterns of histone modifications and TF binding (76) (Figure 1). These patterns modulate the interactions of TFs with chromatin, leading to differences in cell-type–specific transcription (77). They can be identified using chromatin immunoprecipitation (ChIP) coupled with either microarrays (ChIP-chip) or high-throughput sequencing (ChIP-seq). Several studies have identified signatures that are associated with specific genomic contexts and regulatory function (78,79). Many histone modifications are correlated with each other or mutually exclusive, the most obvious example being that two modifications cannot occur at the same position on the tail of the same histone, i.e. H3K27me3 and H3K27ac. However, there are difficulties in determining which modifications are causal and which are the consequence of regulatory binding or transcription without further biochemical studies. These modifications may act in a redundant manner or co-operatively (80), e.g. cross-talk between H3S10ph and H4K16ac has been found to lead to the precise control of transcriptional elongation (81).

We now review what is known about the epigenetic properties of promoters and enhancers, with an emphasis on developmental regulation and associated long-range interactions.

### Chromatin modification states and DNA methylation at promoters

Active promoters are associated with the binding of PolII and TAF1, and their histones are marked with H3K4me3 (76). Results of ChIP experiments have provided further evidence for the different functional types of vertebrate promoters (Figure 1). In most vertebrate genes, the pattern of H3K4me3 deposition closely follows the distribution of CpG islands in the genome (82). In tissue-specific promoters, which commonly lack CpG islands, the H3K4me3 mark is generally limited to the 1–3 nucleosomes downstream of the TSS. In mammals, the H3K4me3 mark extends upstream of the gene start, in agreement with the finding that most CpG island promoters show bidirectional transcription (83–85). Studies in mouse stem cells have revealed that developmentally regulated promoters are associated with a combination of both repressive (H3K27me3) and active (H3K4me3) marks (86). These bivalent promoters are often associated with developmental TFs, which are silenced in embryonic stem cells (ESCs), but they are poised for either rapid activation or inactivation at later stages of differentiation. During this process, the marks associated with these promoters resolve to either repressive or active marks in terminally differentiated cells (87).

DNA methylation in vertebrate genomes can occur at cytosine or adenine nucleotides, but it is found preferentially at CpG dinucleotides. However, in vertebrate genomes, stretches of several hundred base pairs, which are enriched for these dinucleotides, called CpG islands, are frequently unmethylated. CpG islands overlap the majority of promoters, with high-CpG content promoters showing evidence of nucleosome deficiency, transient binding of PolII and small amounts of transcription initiation (88,89). Most regulatory regions, especially promoters, which contain CpG islands are unmethylated. However, during differentiation a number of them become methylated, which is responsible for their committed silencing (90–92). It is thought that methylation is responsible for creating a long-lasting state of repression, which is first preceded by the silencing of gene and deposition of repressive histone modifications, such as H3K27me3 (93). DNA methylation is associated with closed chromatin and is thought to prevent the binding of TFs and PolII to the promoter, thus preventing transcription (94,95). During differentiation, promoters associated with pluripotency factors in ESCs show evidence of preferential methylation (92). However, the majority of promoters that are methylated during differentiation show no evidence of transcription in ESCs and tend to be associated with Type I promoters (96), corresponding to tissue-specific genes not expressed in that specific lineage.

The chromatin and DNA methylation state at most promoters is invariant across different cell types (17). The promoters of developmental genes exhibit the highest level of variability, presumably influenced by regulation from multiple distal and proximal enhancers (97). Constitutively, active genes are typically characterized by an active, open chromatin state and are involved in interactions with a relatively small number of enhancers (98). Type I promoters are only marked by H3K4me3 when the gene is undergoing transcription and are predominantly regulated only by their core and proximal elements (99). Type I and Type III (developmental) promoters show changes in their epigenetic state during differentiation. The latter move from bivalent to an active or inactive state or from active/inactive to inactive/active state. It is unknown whether the changes in the modifications associated with a promoter are a consequence of enhancer:promoter interactions or actually play a role in facilitating them.

### Chromatin modification states at enhancers

Results from ChIP experiments have been useful in identifying putative enhancer elements in the genome. Enhancers are associated with regions of lower nucleosomal density containing specific histone variants, such as H3.3 and H2A.Z (80,100). The presence of these variants contributes to nucleosome instability, allowing TFs to bind more easily and displace nucleosomes (101). These regions are characterized by many different histone modifications and bound by histone-modifying enzymes, such as CREB-binding protein (CBP) and P300 acetyltransferases (102). Enhancers are highly enriched for H3K4me1, H3K4me2 and H3K27ac marks, but they show little or no enrichment for the promoter-associated mark, H3K4me3 (Figure 1). The changes in histone modifications at enhancers play a major role in determining the differential binding of TFs to enhancer elements (77). These patterns are more variable compared with promoters and show good correlation with differences in expression of the target gene (17,103). A study over a large number of cell lines and tissues found that the majority of enhancers show high levels of tissue specificity (104), confirmed by combining the results from ChIP-seq and the mapping of DNase hypersensitive regions (105,106).

Constitutively open enhancers that bind glucocorticoid receptor have been found to be enriched for CpG dinucleotides, whereas enhancers that required chromatin remodelling for activation were not (107). The activation of distal regulatory elements is often accompanied by their demethylation (108,109). Active enhancers that have low levels of H3K27ac and H3K4me1 show higher levels of DNA methylation than enhancers with higher levels (110). These observations reflect the importance of methylation in regulating the cell-type specificity of enhancers and the binding of relevant histone modifiers and TFs.

It is thought that enhancers are initially marked by histone variants and H3K4me1/2 in ESCs, with the binding of P300 and the subsequent addition of H3K27ac leading to their activation. In addition, enhancers can exist in a poised state, marked by H3K4me1 but not H3K27ac. Also, at least a subset of poised developmental enhancers is marked by H3K27me3 (111). Enhancers are believed to be held in this state by Polycomb silencing. In human ESCs, Rada-Iglesias *et al.* (112) identified that active enhancers associated with pluripotency genes, many of which are at large distances from the gene, become inactivated during differentiation, and poised enhancers associated with genes involved in early development become activated. This activation is accomplished by the loss of H3K27me3 and the gain of H3K27ac. Thus, the epigenetic state of an enhancer can provide a measure of its transcription activation potential. Inactive enhancers lack H3K4me2 and have high levels of H3K9me2. In mouse ESCs, Zentner *et al.* (113) found evidence that other classes of enhancer states may exist, potentially specified by additional histone modifications.

The number of enhancer-related histone modifications is still increasing, and as such, the true extent of the enhancer complement of vertebrate genomes is unknown (80,114). Genome-wide profiling of P300, H3K27me3 and H3K27ac in mouse embryonic limb revealed that a significant percentage of experimentally validated enhancers was not identified by either mark (115). This suggests that additional marks play a role in specifying enhancer function and cell specificity. Enhancers containing nucleosomes marked by H4K16ac seem to be able to recruit factors that allow the release of promoter proximal paused PolII (81). H3K8ac is associated with active promoters, but has also found to mark distal elements (80,116) and is a potential indicator of active enhancers.

The epigenetic marks flanking an enhancer are capable of determining its cell-type specificity (117). Investigations of the epigenetic states of the enhancers and promoters of *Mdc* and *IlI2b* in fibroblasts and dendritic cells have found that enhancers flanked by H3K9me3 seem to be unable to drive expression of their target gene, despite being marked by H3K4me1 and being able to drive reporter expression in plasmids. It seems that this mechanism for repressing enhancer activity may be prevalent, as enhancers flanked by H3K9me3 seem to be functional when assayed over a large number of cell types, even though they do not drive expression in their native environment.

## ORGANIZATION OF CHROMATIN AND ENHANCER:PROMOTER INTERACTIONS WITHIN THE NUCLEUS

The spatial organization of chromosomes within the nucleus is a major factor in determining gene expression. Active genes are typically localized in the interior of the nucleus, whereas silenced genes are associated with the peripheral regions. Within the nucleus, chromatin forms several substructures, which are involved in the repression or activation of genes that are co-localized within them.

### Chromosome territories

Each chromosome occupies a distinct chromosomal territory (CT) within the nucleus. In mammalian cells, there is evidence that the relative position of a gene locus within its CT influences its ability to form either *cis* or *trans* interactions. Specific sequences on a given chromosome can loop out of their CT, leading to their subsequent upregulation. Activation of the *HoxB* gene cluster during differentiation coincides with its relocation away from its CT interior (118). Alterations in the localization of a gene with respect to its CT are believed to be controlled by the action of enhancers. The long-range enhancer of the *Shh* gene, required for limb bud development, is important in causing the *Shh* locus to extrude from its CT (119). Within the nucleus, highly expressed co-regulated genes associate together at discrete foci, called transcription factories (120,121). These compartments contain high concentrations of hyperphosphorylated PolII [as well as other TFs (122)], which allows the genes inside them to be efficiently transcribed. Movement of genes into or out of these factories results in their up- or downregulation, and it may explain why genes are transcribed in bursts (123). In some cases, genes found within transcription factories can be located on different chromosomes (124). The movement of genes within the nucleus likely plays a major role in the regulation of gene expression during differentiation.

### Broader chromatin states and relationship with long-range regulation

The human genome shows large regions in which most genes are expressed at high levels, alternating with regions that mainly contain lowly expressed genes. At least half of the *Drosophila* genome consists of multi-gene chromatin domains, which can be large and include dozens of genes (125). These domains might be important for developmental synchronization of genes and are cell-type dependent. Broad regions of histone modifications may reflect both the transcriptional status of these domains and the mechanism by which they are silenced or expressed.

**Figure 2.**
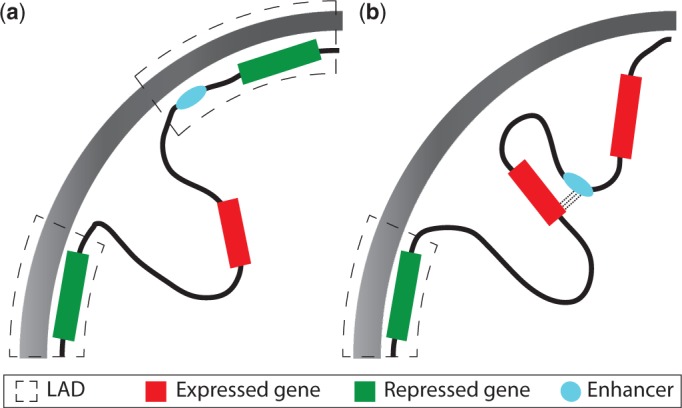
The organization and structure of chromatin within the nucleus is coupled with the regulation of gene expression (125). (**a**) Genes tethered to the nuclear lamina are silenced, whereas genes that are present in the centre of the nucleus are not. In addition, enhancers anchored to the lamina are restricted from interacting with their target promoters. (**b**) The movement of genes away from the lamina results in their upregulation, as they move to a more permissive transcriptional domain, and the movement of enhancers allows them to communicate properly and regulate the expression profile of their target gene.

Large domains of chromatin (0.1–10 Mb) have been found to be in contact with the nuclear lamina (126) (Figure 2). These lamina-associated domains (LADs) are transcriptionally inactive, show enrichment for H3K9me2 and are depleted in active histone modifications. Large changes in the levels of PolII and H3K4me2 were observed at the boundaries of LADs. The interaction of a gene with the lamina directly leads to the silencing of transcription (127). Large domains of H3K9me2 (128), called LOCKS, overlap with LADs. These domains show changes during differentiation and are thought to be cell-type specific. This indicates that chromatin differentially associates with the lamina depending on the cell type. Genes that are not expressed in a specific cell type may be tethered to the lamina, whereas genes expressed in that specific cell type are not. This reorganization of the genome in the nucleus seems to be responsible for loss of competence during neuronal differentiation in *Drosophila* (129). The hunchback locus, which is responsible for determining cell fate, is first downregulated and then relocates towards the lamina leading to its permanent repression. During differentiation, genes that move away from the lamina are activated, as they become localized to the interior regions of the nucleus.

Studies have shown that Polycomb and H3K27me3 can form continuous chromatin domains, which can be larger than 100 kb and show overlap with silenced genes and intergenic regions (130). These domains extensively and specifically interact with each other, with the majority of interactions between regions on the same chromosomal arm (131,132). Long-range contacts with sites devoid of Polycomb binding were found to be rare.

## FACTORS INVOLVED IN MEDIATING LONG-RANGE INTERACTIONS

The mechanisms that modulate long-range interactions are currently poorly understood. Factors such as CTCF, cohesin, mediator and small RNAs (133) seem to play important roles in this process. Mediator and cohesin have been found to co-occur together at the promoters of active genes (134). In addition, there is evidence that a direct interaction between PolII and mediator is required for transcription (135), and that interactions between paused PolII and cohesin are required for transcriptional elongation to occur (136).

Regulatory elements referred to as insulators are able to block communication between enhancers and their cognate promoter. These elements are thought to function by blocking enhancer:promoter interactions (137), either by the insulator element binding to the enhancer or promoter and preventing them from interacting, or by partitioning the genome in a series of loop-like structures such that elements in one loop are not able to interact with elements in a different loop (138). CTCF, which binds at insulators, has been found to be involved in promoting and mediating long-range enhancer:promoter interactions (139), and it may be responsible for demarcating cell-type–specific regulatory regions (140). Along with CTCF, cohesin is also thought to play a major role in controlling 3D conformation (41). Recent work has proposed that CTCF may also play a role in regulating relatively short-range enhancer:promoter interactions (on the order of kilobases) (16) by altering local nucleosome configuration (141), whereas additional factors are required for long-distance communication.

It is suggested that CTCF, cohesin and mediator may be involved generally in the formation of chromatin structures, whereas specific TFs and their coactivators may be involved in controlling locus-specific looping interactions and structure. Through the use of a novel tethering assay, Deng *et al.* (142) identified that *Ldb1* is an important TF involved in *GATA-1*-mediated looping in the β-globin locus. *Ldb1* was attached to a zinc-finger protein, which allowed it to be artificially anchored to specific regions within the β-globin locus. In the absence of *GATA-1*, transcription of β-globin could be induced by attaching this construct to its promoter. This region was found to directly interact with elements at the locus control region, suggesting important roles for this factor in regulating long-range interactions.

Several studies have found that non-coding RNAs (ncRNAs) are important in mediating enhancer:promoter interactions. *HOTTIP* is a long intergenic non-coding RNA (lincRNA) located at the 5′ section of the *HOXA* locus (143), which is required for coordination of several distal HOX genes and is required for the maintenance of H3K4me3 at these promoters. It seems that looping brings *HOTTIP* into close proximity with its target genes and recruits *WDR5-MLL* to the locus. Knockdown of this lincRNA by siRNA does not alter the higher order structure of this locus; however, there is a reduction in the level of promoter-associated H3K4me3. A subset of ncRNAs called *ncRNA-a* (ncRNA-activating) seems to work by activating their neighbouring gene in *cis*. A number of these ncRNAs seem to bind with mediator, resulting in the expression of their target genes (144). Depletion of either the reported ncRNA-a transcripts or mediator subunits reduced looping between target genes and their regulatory elements, but did not completely remove it. This suggests that at least a subset of ncRNAs involved in long-range interactions work by stabilizing pre-existing chromatin structures rather than being directly involved in their formation.

## ENHANCER TRANSCRIPTION AND ITS ROLE IN LONG-RANGE TRANSCRIPTIONAL REGULATION

Although transcription at enhancers was first observed >20 years ago (38), only recently has evidence been found that this phenomenon is both widespread and indicative of enhancer activation. In mouse motor neurons, Kim *et al.* (145) found that the transcriptional co-activator CBP recruits PolII to enhancers, leading to the bidirectional transcription of ncRNAs called enhancer RNAs (eRNAs). The level of eRNA expression was found to correlate well with expression at nearby genes. These eRNAs are produced bidirectionally from within a relatively small region (<2 kb), and not over the entire distance between the enhancer and promoter, providing further evidence that the tracking model of enhancer:promoter communication is incorrect. Transcription of eRNA did not occur unless there was a direct interaction between an enhancer and its cognate promoter, leading to the proposition that the PolII, which binds at an enhancer is transferred to its target promoter via looping. Analysis of Hi-C data generated by the ENCODE consortium has also found that enhancers involved in physical interactions are significantly more likely to be associated with transcription of eRNA (27).

In macrophages, a significant amount of PolII binding occurs extragenically (146), with 70% of these binding events located within putative enhancer regions. Transcription was confirmed at a number of these enhancers using quantitative polymerase chain reaction, and unlike the eRNAs reported by Kim *et al.*, these transcripts were found to be polyadenylated. This study found that the number of non-transcribed enhancers was considerably larger than the number of transcribed ones, suggesting that they could belong to two different groups. Inhibition of RNA synthesis resulted in decreased acetylation at both the TSS and upstream regions.

Wang *et al.* (147) found that eRNA expression was better at indicating enhancer activation than TF binding and histone modification data. In addition to chromatin state and evolutionary conservation, the detection of eRNAs has been used to identify putative enhancers (148). It remains an open question whether these eRNAs are simply transcriptional noise or are functionally important. Recently, eRNAs produced by enhancers bound by p53 have been found to directly enhance transcription at multiple distant genes (149); siRNA knockdown of a number of these eRNAs resulted in a decrease in expression of their target genes, while maintaining existing chromatin interactions. Other classes of ncRNAs stabilize long-range enhancer:promoter interactions (150) or recruit chromatin remodellers (151), and eRNAs might perform a similar role.

## DIRECT 3D INTERACTION INSIGHTS ON PROMOTER–ENHANCER INTERACTION

A single promoter can be involved in interactions with a single or multiple enhancers (152). Enhancers can either interact with a specific target gene or with many genes (153) (allowing the coordinated regulation of functionally related genes). Type I (tissue-specific) PolII promoters are typically controlled by regulatory elements in close proximity (99), whereas Type III (developmental) promoters are most often controlled by long-range regulation (13).

Evidence suggesting that physical interactions between enhancers and promoters are necessary has been found using a variety of approaches, including fluorescence *in situ* hybridisation (FISH) and chromosome conformation capture methods. By tagging specific sequences with fluorescent probes, FISH allows the identification of regions of the genome that are brought into close spatial proximity (154). Chromosome conformation capture methods (155–158) are useful for assessing the frequency of interactions between two genomic loci: either between two pre-selected loci (3C), one locus and the rest of the genome (4C, 3C-seq) or all interactions between multiple pre-selected elements (5C). Hi-C allows unbiased and genome-wide investigation of interactions, although currently its resolution is relatively low compared with other techniques (159,160). ChIA-PET (chromatin interaction assay with paired-end sequencing) makes it possible to identify all interactions mediated by a specific protein, allowing investigation of how specific TFs (and histone modifications) alter the structure of chromatin (161). Currently, the available evidence from 3C-based methods is suggestive of direct physical interactions between enhancers and promoters. However, recent work has identified that the results from FISH and different 3C-based methods are not always concordant (162), e.g. many of the loci predicted to interact by 5C were not found to co-localize in FISH experiments. The suggested reasons for these observations are that FISH is a more disruptive method leading to loss of some interactions during cell treatment, or that formaldehyde cross-linking in 3C-based methods does not always reflect average spatial distances between the interacting loci. Nevertheless, these technologies have provided insights into how promoters and enhancers communicate in 3D space and into the effects of chromosomal conformation on gene expression (159).

### Locus control regions and active chromatin hubs

**Figure 3.**
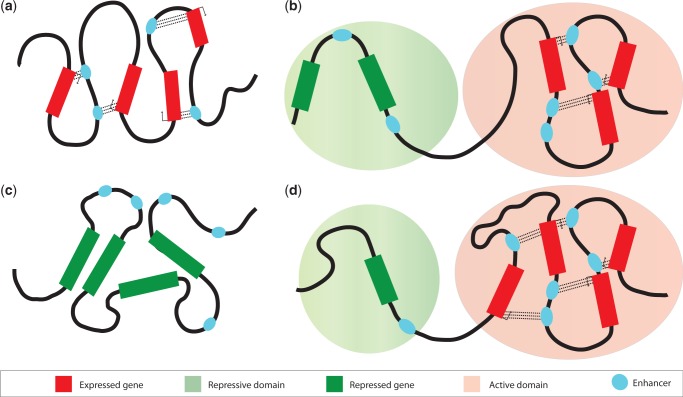
Chromatin looping is responsible for forming higher-order hub-like structures within the nucleus. (**a**) An active chromatin hub (ACH) is a structure that allows enhancers and promoters to come into close spatial proximity with each other (40). This structure has a high concentration of chromatin remodellers and PolII, which allows stable/high levels of transcription. Interactions between promoters and enhancers and represented by dashed lines. (**b**) The recruitment of genes and enhancers to a repressive chromatin hub (RCH) results in their downregulation (165). This structure potentially prevents enhancers from communicating with their cognate promoter by looping them out and preventing them from interacting. This structure may also restrict the amount of PolII from binding to gene promoters. (**c** and **d**) During development, genes in the Hox locus show a linear movement from a repressive chromatin structure to a region where they are expressed (166). This movement allows enhancers to interact with targets that were previously held in the repressive domain.

In erythoid cells, where β-globin is expressed, these interactions form a compartment containing regulatory elements that has a high level of transcriptional activity (40). These chromatin structures depend on sequence-specific TFs bound to both the enhancer and promoter and thus can explain the specificity observed in enhancer:promoter interactions. Knock out of TFs responsible for regulating β-globin expression (*EKLF* and *GATA-1*) result in the loss of interactions between the gene and its enhancers, leading to loss of the overall hub-like structure (163,164). Interactions between regulatory elements separated by large genomic distances have been observed at high frequencies at other loci, leading to the proposition that chromatin looping generally results in the formation of hub-like structures. These active chromatin hubs (ACH) are responsible for bringing enhancers and promoters into close spatial proximity as well as providing an environment that is transcriptionally permissive (Figure 3a). The contents of the β-globin ACH changes during differentiation, with the ACH lacking certain elements in progenitor cells, which are found there later during differentiation (167). At the *Myb* locus during erythoid proliferation, intergenic enhancers, the *Myb* promoter and its first intron are brought together to form an ACH (168). The ACH leads to high concentrations of PolII and TFs being present around *Myb*. The first intron of this gene contains a site for regulating transcriptional elongation. Interactions between this element and distal enhancers lead to the generation of full-length transcripts. This structure is lost when cells terminally differentiate, coincident with the loss of expression of *Myb* and a reduction in TF binding at regulatory elements.

### Chromatin can form structures that prevent enhancer: promoter communication and repress transcription

Chromatin hub-like structures have also been found to play a role in repressing gene expression (165). Studies of the *GATA-4* locus in undifferentiated Tera-2 cells have revealed the existence of a 3D structure consisting of multiple chromatin loops (Figure 3b), termed a pre-repressive chromatin hub (pre-RCH), which is lost when these cells differentiate. This structure was found to comprise several H3K27me3-enriched elements and was maintained by Polycomb, suggesting that Polycomb may repress genes through the formation and maintenance of these structures. In undifferentiated cells, this structure results in *GATA-4* being held in a poised state, and its loss during differentiation was accompanied with an increase in the expression of *GATA-4*. This structure may function by either preventing enhancer:promoter interactions or by restricting the access of PolII to the *GATA-4* promoter.

### Coordinated developmental control of Hox loci by long-range regulation

In vertebrates, Hox genes are typically organized into clusters and are transcribed sequentially; with the first gene in the cluster being expressed in the anterior part of the organism. This collinear expression pattern results from changes in both the histone modification patterns within a Hox cluster and its overall 3D conformation (118). Within a Hox cluster, active and inactive genes are separated into distinct domains labelled by distinct histone modifications, H3K4me3 and H3K27me3, respectively. It is thought that Hox genes are expressed sequentially because of their highly regulated movement out of chromosome territories and the decondensation of associated chromatin (169). Studies of the *HoxD* locus using 4C (166) have found that when all genes within a cluster are not expressed, these genes form a single 3D structure that represses transcription. This led the authors to propose a model whereby as genes are expressed they progressively migrate from this structure and cluster into a transcriptionally active structure, leading to a bimodal organization of chromatin at a single Hox cluster (Figure 3c and d). This movement is associated with changes in histone modifications and suggests that the observed collinear expression may be the result of a stepwise movement of genes from an RCH-like structure to an ACH-like structure.

An examination of the HoxD locus, important for limb development, using chromosome conformation capture techniques has revealed that this locus has a tissue-specific conformation involving interactions between genes and enhancers located within the adjacent gene deserts (152). The active section of gene cluster seems to be in contact with several enhancers concurrently, each of which seem to be important for some aspect of limb development. The concept of a regulatory archipelago proposed by Montavon *et al.* (152) is basically the same as the previously proposed GRB model of transcriptional regulation (9). These findings suggests that the 3D conformation of loci changes during differentiation as a result of interactions between promoters and enhancers, resulting in the creation of cell-type–specific patterns of gene expression and organization. Indeed, the most recent results suggest that the 3D conformation at developmental loci is both the most dynamic and most divergent across mammals (170).

### Hi-C provides information on the global patterns of long-range interactions within the genome

Hi-C has provided further evidence for the presence of CTs and found that eukaryotic genomes are organized into functional domains (A and B compartments) that are important for controlling DNA transcription (159). By applying a hidden Markov model to Hi-C data, Dixon *et al.* (160) were able to partition the genome into megabase-sized domains of chromatin, called topological domains. Elements within these domains preferentially interacted with other elements in the same domain. The boundaries of these domains were found to be associated with known elements displaying barrier activity and correlated well with known CTCF-binding sites.

Hi-C has enabled the calculation of the probability that two randomly chosen loci interact (159). This contact probability follows a power-law distribution and suggests that chromatin is organized globally in a fractal globule structure. However, the exponent that describes this distribution has been found to vary (171), and recent studies have found that the contact probability plateaus as the genomic distance increases (172). These findings suggest that this model cannot adequately describe the observed patterns of chromatin folding. The strings and binders model (173) has been proposed, which not only recapitulates this structure but is also more related to the known underlying biology of protein-mediated chromatin folding.

### 5C confirms non-linear arrangement of enhancers and their target genes

By examining 5C data from three different cell lines, Sanyal *et al.* (27) found evidence that large numbers of looping interactions were cell-type specific and tended to occur between active functional elements. Long-range interactions between enhancer:promoter pairs were found in domains enriched for both H3K9ac and H3K27ac. Additionally, only 7% of identified interactions were between a distal element and it’s nearest TSS (this increased to 22% when only considering active TSSs), which shows the flaw in studies that assign enhancers and other distal elements to the nearest TSS.

### Chromatin folding mediated by transcription factors reveals an extensive network of interactions between genomic elements

ChIA-PET has revealed insights into how these interactions are mediated by TFs, allowing the generation of genome-wide chromatin interactomes. In the initial ChIA-PET experiments, estrogen-receptor (ER)-α was used as bait to identify interactions between regions of the genome, which it was mediating (161). As expected, most interactions occur between elements located on the same chromosome, rather than interchromosomally. A positive association was found between chromatin interactions and the activation of genes involved in those interactions. In addition to identifying interactions between distal sites and promoters, a number of interactions were found between distal sites, hinting at the possibility of widespread enhancer:enhancer interactions. Li *et al.* (154) used PolII as bait in ChIA-PET experiments and identified that promoters are involved in three distinct types of interactions; either with the body of the gene, with distal elements or with other promoters. Expression of genes regulated by promoters involved in interactions with other promoters was found to be highly correlated. This may explain how transcription of tissue-specific and housekeeping genes is co-ordinated. Long-range interactions between distal elements and promoters were found to be highly cell-type specific, and promoters were involved in interactions at different distances depending on the cell line under investigation. To investigate enhancer:promoter interactions, Chepelev *et al.* (174) used the enhancer mark H3K4me2 as bait and identified >6000 potential interactions. Promoters that were found to interact with the same enhancer showed evidence of tissue-specific co-expression.

ChIA-PET has also revealed that CTCF-mediated interactions correlate with distinct domains of histone modifications in the genome (175). The interactions between CTCF-bound sites and promoters seem to be more cell-type invariant when compared with enhancer:promoter interactions, as is CTCF binding. As such, insulators involved in enhancer-blocking may function to mediate cell-type–specific long-range interactions. However, recently a number of looping interactions were found to skip sites bound by both CTCF and cohesin, which may suggest that additional factors are required (27).

This form of interaction data can be easily represented as a network, with an edge present between two nodes (each of which corresponds to a region of chromatin), when they have been found to interact. A large connected component (containing ∼40% of the elements involved) has been identified within the network of PolII-mediated chromatin interactions (176). This network exhibits a hierarchical structure and has a scale-free degree distribution. Investigation of communities of strongly connected elements within this network revealed that these were involved in functional compartmentalization. The majority of these chromatin communities were conserved between cell lines, indicating that cell-type specificity may be defined by long-range transient interactions or by small differences in the content and organization of these communities.

## CONCLUSION

The identification of the different factors and histone modifications involved in long-range regulation will help elucidate how specific enhancers target specific promoters without binding to intervening promoters.

It does not seem that there are any features specific to enhancers involved in long-range regulation compared with those involved with regulation over shorter distances. Indeed, it seems that the ability to respond to long-range interactions depends on the promoter architecture of a gene and the state of intervening insulators. The development of new genome-wide techniques has provided a way to systematically identify regions that are targeted by specific proteins and co-localize in nuclear space. The results from ChIP-seq and chromosome conformation capture assays are, however, averaged over a cell population, and they do not provide any information on the temporal dynamics and cell-to-cell variation of long-range interactions. Improvements in the temporal resolution of experiment techniques should help determine how chromatin moves during the cell cycle and how this affects and which genes are upregulated and downregulated at specific stages. Studies of long-range interactions are also limited by the relatively low resolution of Hi-C. As this improves, we expect that new findings will follow regarding the global patterns of interactions between individual regulatory elements. Examination of this in multiple cell types and species will enable us to understand how the constraints on 3D conformation affect the arrangement and conservation of regulatory elements.
